# The Principles and Practice of Obstetric Medicine and Surgery, in Reference to the Process of Parturition: Illustrated by 142 Figures

**Published:** 1842-04

**Authors:** 


Art. V.—*The Principles and Practice of Obstetric Medicine and Surgery, in reference to the Process of Parturition: Illustrated by* 142 *figures.* By Francis H. Ramsbotham, M. D., Consulting Physician in Obstetric Cases to, and Lecturer on Obstetric and Forensic Medicine at, the London Hospital, Physician to the Royal Maternity Charity, Obstetric Physician to the Eastern and Tower Hamlets' Dispensaries. First American edition, revised: pp. 458, large octavo. Lea & Blanchard, Philadelphia, 1842.

The author of this work is the son of a celebrated English obstetrician and writer on obstetrics, and is himself already favorably known by his lectures and writings upon this department of medical science, the former of which were given to the profession through the London Medical Gazette, in 1833–4. He has also, we believe, published an "Atlas of Midwifery." Having the immediate benefit of the vast experience of his father, joined to that acquired by himself in an extensive public and private practice, he could not fail to produce a highly valuable work, such, in fact, as we conceive the one before us to be.

The present treatise is more particularly designed for students, and seems admirably adapted to their wants. It is highly practical; the author introduces nothing that does not strictly appertain to his subject, and avoids those long disquisitions on minor points that detract greatly from some of the more popular works on midwifery. It is written in a clear and perspicuous style, in which respect, as in the one just mentioned, it offers a remarkable contrast to the treatise of our countryman, the late Dr. Dewees. The latter, though one of great value, and of more authority, perhaps, than any other medical book ever issued from the press in this country, is certainly obnoxious on the score of style, and on account of the tiresome discussions into which it enters.

In the first portion of his work, Dr. Ramsbotham gives us the anatomy of the healthy and deformed pelvis, the shape and dimensions of the fœtal head at birth, the structure of the female generative organs, and the description of the gravid uterus. These subjects take up about 90 pages, and are illustrated by numerous lithographs. In his statement of the difference in form between the male and female pelvis, we notice an error, which, however, is a very common one. He describes the sacrum as being much straighter in the male than in the female skeleton, whereas the fact is directly the reverse, as may be seen by comparing the two together.

The remainder of the work is devoted to the subject of labor, which he divides, like Denman, into four classes: 1st. Natural, 2nd. Difficult. 3d. Preternatural, 4th. Complex; and under these various heads are discussed some of the most important questions that demand the attention of the physician or surgeon accoucheur.

The first class, or NATURAL LABOR, occupies about sixty pages, wherein the process is fully and minutely explained, being traced step by step from its commencement to its final termination, or the expulsion of the placenta, at the same time that ample details are given touching its management, and also respecting the conduct and duties of the accoucheur during its progress and subsequent to its completion. All this is illustrated, wherever practicable, by beautiful and accurate engravings. We would dissent from but one thing in this portion of the treatise, and that is, the arrangement of head presentations into eight varieties; too much refinement in this respect always tends to defeat the very object for which it is proposed, and to perplex rather than enlighten the student.

The second class of labors, DIFFICULT or LABORIOUS, embraces two orders, the *lingering* and *instrumental.* The causes of the former are discussed at length under two heads—those referable to the mother—and those in which the ovum is at fault. Instrumental labors, the second order, are subdivided, first, into those accomplished by instruments perfectly compatible with the life of the child, and the safety of the mother's structures; and, secondly, those in which either the child must be sacrificed, or a cutting operation performed on the mother's person. Under these two subdivisions, he treats of the forceps, both long and short, their application and the indications for their use; of the vectis, and the relative advantages of this instrument and the forceps; of craniotomy, the cases requiring it, and the instruments necessary to its performance; of the Cæsarian and Sigaultean operations; and lastly, of the induction of premature labor. He does not fail constantly to impress on the mind of the young practitioner that nothing short of the most urgent necessity will warrant him in taking an obstetric instrument in hand, and that in no case is such aid to be thought of, save as a last and only resort. As to forceps, he prefers the straight ones of Denman, and these he recommends to his junior brethren, at least in the commencement of their practice, in preference to those with Leveret's, or any other lateral curve.

In speaking of the induction of premature labor he notices a mode of practice, which, as it is of recent introduction, may be new to some of our readers. After describing the methods in use, he says:

"Being impressed with the great advantage of preserving the membranes whole, I made some experiments with a medicine (ergot) now well known, and found expulsive action soon follow its exhibition in all the instances, with very few exceptions, where I administered it.

After a great number of trials, however, I observed that, although the mothers recovered as well as if they had gone through an ordinary labor, their systems not being in any sensible degree injuriously affected by the drug, (and in some instances between thirty and forty doses were taken,) yet that the proportion of children born still was greater than when the membranes were punctured. This I attributed to the baneful influence of the medicine upon the fœtus. I was consequently led to modify the practice; and I am now in the habit of administering four or five doses at intervals of four or six hours, and of rupturing the membranes after their exhibition. I have generally remarked that the os uteri has become soft, and mostly somewhat opened under the action of the drug; and the increase of live births has been larger when this system was followed, than after any other which I have tried."

The third class, or PRETERNATURAL LABOR, likewise embraces two orders: first, those in which the breech or any part of the lower extremities presents; second, those in which the child offers itself transversely—*cross-births* properly so called. The various presentations, with the management they require, are carefully described, in the comprehension of which the student is greatly aided by the figures that accompany this portion of the work.

COMPLEX LABORS, the fourth and last class, comprise all not included in the former, and are divided into ten orders: 1st. labors complicated with hemorrhage; 2nd. with convulsions; 3d. with ruptured uterus; 4th. with lacerated vagina; 5th. with ruptured bladder; 6th. with syncope, independently of hemorrhage, or any extensive laceration or other lesion; 7th. with descent of the funis by the side of the head, or breech; 8th. with descent of the hand by the head or breech; 9th. labors in which monsters are produced; and 10th. in which there is a plurality of children.

The reader will not fail to observe that cases are here grouped together, differing widely from each other in their nature, and requiring great diversity in their management. Yet, for purposes of instruction, this arrangement is perhaps as good as any other; a multiplication of classes would be both inconvenient and perplexing. In this part of the work, as throughout the whole, the principles and rules of action, such as they have been deduced from the largest experience, and sanctioned by the best authorities, are distinctly and clearly set forth.

We have thus given a hasty synopsis of the various subjects treated of in this volume, in lieu of a regular analysis of its contents, for which we have not space at present. We do not hesitate to recommend it—the book, we mean—to our readers, believing it to be, for elementary instruction at least, a most excellent one. To the student, such a work would seem indispensable; and we may be permitted to add, that if, with its lucid text, and admirable illustrations, he should fail to acquire a respectable knowledge of the principles and practice of obstetric medicine and surgery, he had better give up the attempt in despair. C.

